# The “Aberdeen Home Continence Stress Test”: a novel objective assessment tool for female stress urinary incontinence

**DOI:** 10.1007/s00192-023-05530-4

**Published:** 2023-04-13

**Authors:** Catriona Young, David Cooper, Alyaa Mostafa, Mohamed Abdel-Fattah

**Affiliations:** 1 The Aberdeen Centre for Women’s Health Research, University of Aberdeen, Foresterhill, AB25 2ZD Aberdeen, UK; 2 Health Services Research Unit, University of Aberdeen, Aberdeen, UK

**Keywords:** Incontinence, Stress incontinence, Diagnostic accuracy, Measurement tool

## Abstract

**Introduction and hypothesis:**

Clinical trials for stress urinary incontinence (SUI) require a robust, reliable, and responsive tool for objective assessment of SUI post-intervention. The Aberdeen Home Continence Stress Test (HCST) is a novel patient-reported objective assessment tool, aimed to be patient-friendly and reduce attrition rates by avoiding hospital appointments and prolonged pad-wearing. We aim to describe the HCST for the first time and evaluate its reliability, diagnostic accuracy, and response to change.

**Methods:**

A secondary analysis of the Single-Incision Mini-Slings (SIMS) study (a prospective multicentre randomised control trial (RCT) comparing two surgical treatments of SUI was performed. In SIMS (*n* = 600 women), the objective outcome was assessed by the 24-h pad test, while the patient-reported success rates were assessed using the Patient Global Impression of Improvement (PGI-I) at 15 months, 2 years and 3 years post-randomisation. Participants were instructed to perform the HCST before and after the 24-h pad test. The HCST was analysed in relation to reliability, validity, and the relationship between the 24-h pad test and HCST results and finally with regard to its responsiveness to change in PGI-I. (Trial registration-number ISRCTN93264234, registration date 14/01/2014).

**Results:**

Compared to the 24-h pad test, the sensitivity of the HCST ranged from 0.81–0.95, specificity was 0.76–0.79, negative predictive value was 0.96–0.99 and positive predictive value was 0.32–0.43. Reliability was indicated by high-performing Cronbach’s alpha value (> 0.7). An improvement of ≥ 2 leakage groups on the HCST (for example from Large at baseline to Small leakage at follow-up) was strongly associated with patient-reported success on PGI-I (OR 4.38, 95% CI 2.31, 8.31).

**Conclusions:**

The HCST is a valid and reliable patient-reported objective assessment tool that can be used for assessing SUI in surgical trials with good specificity, sensitivity, and consistency.

**Supplementary information:**

The online version contains supplementary material available at 10.1007/s00192-023-05530-4.

## Introduction

### The need for a novel tool

Stress urinary incontinence (SUI) refers to a complaint of involuntary loss of urine on effort or physical exertion [1]. It is both a common and debilitating condition affecting one in four women [1]. The combination of its high prevalence, impact on women’s quality of life (QoL), and burden to health resources makes SUI an important field of research [2, 3]. A robust, reliable, and responsive assessment tool is essential for the evaluation of SUI interventions. The tool must also be participant friendly to reduce attrition rates and consequently improve efficiency in clinical trials.

Currently, urodynamics testing is the ‘standard’ investigation in assessing SUI in clinical practice [4]. However, its invasive nature, associated patient embarrassment, cost to health resources, and potential risk of lower urinary tract infections (UTIs) limits its use in research [5, 6]. Similar limiting obstacles arise with the current alternatives.

A common non-invasive test is the clinician-observed cough stress test (CST). The CST is a common office-based test in which a health professional reports any leakage from patients following coughs in a supine or standing position. Despite common use in clinical practice, it is also burdened by standardisation difficulties [7], and the need for a clinic appointment, which incurs the cost of healthcare resources and the time/travel costs to the participant. Independent of these human and financial factors, the current evidence supports the validity of the CST [7].

Another common test used for SUI assessment is the pad test, where the patient wears a pad for a specified period of time before giving it back to researchers to record the weight gain. There is a range of intervals at which patients are asked to wear the pads: 1, 2, 8, 12, 24, and 72 h [8]. Those studies relying on the shorter tests (1, 2 and 8 hours) have the benefit of better standardization [3], at the cost of poor reliability [8, 9]. While the longer test (24 h) is validated with respect to both reliability and validity [3, 8, 10]. the 24-h pad test is neither invasive nor requires a clinic appointment. However, participants in a recent study [11] reported the 24-h pad test to be the least favored, second only to urodynamic testing. Studies have used varying 24-h pad gain for a diagnostic threshold, with many accepting > 8 g as positive test [2, 12], while other studies reported 1.3 g, 1.4 g, 4 g, and 5 g as diagnostic [13–16].

Both the CST and the 24-h pad test demonstrate favourable reliability and validity [17]. However, the practicalities of these tests make them cumbersome and less preferred both from the individuals’ perspectives and for their use of healthcare resources [18].

Clinical trials are the cornerstone of evidenced-based medicine. There is a growing interest in how best to recruit and retain participants to both preserve the quality of research and to keep within budget and achieve deadline targets [19]. The hypothesis of this study is that the Aberdeen Home Continence Stress Test (HCST) will be demonstrated as a valid patient-friendly objective assessment tool for assessment of SUI post-intervention in clinical trials.

### Aim

The aim of this project was to compare the HCST to the 24-h pad test in its ability to detect SUI. This study approached the aim by addressing the following objectives:

1. To describe the HCST as an assessment tool for SUI.
2. To assess the consistency of the HCST responses.
3. To assess the diagnostic accuracy of HCST in comparison with the 24-h pad test.
4. To compare the responsiveness to change of the HCST by comparing to the outcome on the Patient Global Impression of Improvement (PGI-I).

## Materials and methods

### Study population

This study was a secondary analysis from the Single-Incision Mini-Slings (SIMS) study, a prospective multicentre randomised control trial (RCT) of mini-slings compared to standard mid-urethral slings in the surgical treatment of SUI in women [20]. The SIMS Study recruited 600 women from 21 hospitals in the UK between February 2014 and September 2017 [20]. After consultation of the exclusion criteria, 596 women remained for analysis (see Appendix B1). The inclusion criteria were women 18 years and older, with SUI, who failed or declined conservative management and intended to receive a mid-urethral sling. The exclusion criteria were anterior or apical prolapse of stage two or higher previous SUI surgery, mixed urinary incontinence (MUI) with predominant urgency incontinence, planned concomitant surgery, previous pelvic irradiation, pregnancy or planning pregnancy, and inability to consent in English. Data were collected at baseline, and at 15, 24, and 36 months post-randomisation. Ethical approval was granted by North of Scotland Research Ethics Committee as pertaining to the SIMS trial [20]. This study was reported in accordance with the STARD checklist [21].

### The Aberdeen Home Continence Stress Test (HCST)

Participants were instructed to drink fluids until the sensation of bladder fullness and normal desire to void. In the convenience of their own homes, they stood with feet shoulder-width apart and undressed from the waist down over a pre-provided paper-tissue sheet. Then they coughed loudly (three sets — each of three coughs) in short succession in order to trigger the Valsalva maneuver. The participants then responded to: Question one) whether leakage appeared on the sheet, Yes/No (positive/negative value), Question two) how much leakage appeared, None/Small/Moderate/Large according to a schematic diagram (see Appendix B2). The instructions that participants received appears in Appendix B3.

All participants were asked to complete the objective assessment pack at baseline. This included performing the HCST twice — 24 h apart before and after undertaking the 24-h pad test. At each follow-up point, only participants who returned the patient-reported assessment pack were sent the objective assessment pack The patient-reported assessment at follow-up included the PGI-I scale [22]. The PGI-I is a subjective seven-point scale of the participant's perception of symptoms following an intervention with the following options: Very much better = 1, Much better = 2, Little better = 3, No change = 4, Little worse = 5, Much worse = 6, Very much worse = 7. Previous literature supports the PGI-I as a validated measurement of patient-reported outcomes [22, 23].

### Data analysis

Diagnostic accuracy was the primary outcome. This was assessed by comparing the negative/positive values of the HCST to that of the 24-h pad test to identify the sensitivity, specificity, positive predictive value (PPV), and negative predictive value (NPV). The secondary outcomes included reliability, association to the 24-h pad test values, and response to change.

The reliability of the HCST was assessed by testing the consistency of the HCST results performed 24 h apart. The HCST was analysed by using Cronbach’s alpha between both the positive/negative value of the HCST and the leakage amount questions that were completed 24 h apart. Cronbach’s alpha is an estimation of consistency of items within a measure extending from zero to one. The higher the value, the greater the consistency [24]. 0.7 was predetermined at the minimum threshold according to the literature [25].

A linear regression model was created to explore the relationship of the pad weights and the severity of leakage on HCST (grouped as None, Small, Moderate, and Severe) using an unadjusted model followed by an adjusted model. Adjustments were included for age, body mass index (BMI), parity, pelvic floor management training (PFMT), and previous gynaecological surgery. Subgroup analysis was also performed to consider if the effects change with (a) severity of urgency and (b) diagnosis of MUI.

PGI-I responses were analysed using logistic regression to examine response to change. The HCST responses were categorized as: (a) Gets worse, b) No change, c) Small improvement (reduction in leakage at follow-up compared to baseline by one group for example from Moderate to Small on the HCST diagram), or d) Big improvement (reduction in leakage at follow-up compared to baseline by ≥ 2 groups — for example, from Moderate to None on the HCST diagram). The PGI-I responses were grouped as Very much better/Much better as success and all other responses treated as failure This is how the primary outcome was defined in the SIMS trial [17].

Repeated observations were adjusted for using a random effect (intercept) in all necessary models. Analysis was performed using the IBM Corporation Statistical Package for the Social Sciences (SPSS) for Windows version 27 [26], with the exception of Excel Workbook for diagnostic accuracy. The data management and cleaning used is described in Appendix B4. The first HCST responses were used for the analysis (except for the comparison in Cronbach’s alpha that used both).

A post-hoc sample size calculation on the precision of the confidence interval around the estimated sensitivity or specificity was calculated, and with 443 participants, there is 90% power that the maximum width of the confidence interval around the estimated diagnostic accuracy measure is 0.1319.

## Results

### Characteristics of the participants

Of the 596 participants in the SIMS RCT [20], 443 participants performed the HCST at baseline, 229 at 15 months, 215 at 2 years, and 176 at 3 years.

Table 1 compares the characteristics of participants who performed the HCST at baseline to those who did not. The characteristics were similar with the exceptions of smoking status (14.7% HCST vs 20.4% no HCST), current use of anti-cholinergics (17.6% HCST vs 11.1% no HCST), and those who previously received gynecological surgery (99.8% HCST vs 62.1% no HCST).

**Table 1:** Characteristics of the participants at baseline according to whether the HCST was performed

		Performed HCST	No HCST performance
		(*n* = 443)	(*n* = 153)
Characteristics		Median/mean/count (IQR/SD/percentage)	Median/count (IQR/percentage)
Age	Median (IQR^a^)	49 (43, 57)	47 (43, 56)
BMI	Median (IQR^a^)	28 (25, 32)	27 (25, 32)
ICIQ-UI-SF^c^	Mean (SD^b^)	14 (3)	14 (3)
Number of pregnancies	0	13 (2.9%)	6 (4.1%)
	1	61 (13.8%)	15 (10.2%)
	2	196 (44.2%)	64 (43.5%)
	3	111 (25.1%)	51 (34.7%)
	≥ 4	62 (14.0%)	11 (7.5%)
Current smoking status		65 (14.7%)	30 (20.4%)
Manual (heavy lifting) job		126 (28.5%)	42 (29.2%)
Pelvic floor management training		380 (85.8%)	128 (83.7%)
Currently uses anti-cholinergic drugs		78 (17.6%)	17 (11.1%)
Used previous anti-cholinergic drugs		76 (17.2%)	23 (15.0%)
Urgency perception	None	64 (14.4%)	25 (17.7%)
	Mild	124 (28.0%)	41 (29.1%)
	Moderate	183 (41.3%)	49 (34.8%)
	Severe	68 (15.3%)	26 (18.4%)
	Missing	4 (0.9%)	0 (0.0%)
Urodynamic diagnosis	Urodynamic stress incontinence	348 (78.6%)	118 (77.1%)
	Urodynamic mixed incontinence^d^	60 (13.5%)	9 (5.9%)
	Equivocal	5 (1.1%)	1 (0.7%)
	Not interpretable	1 (0.2%)	1 (0.7%)
	Other	4 (0.9%)	0 (0.0%)
	Missing	5 (1.1%)	19 (12.4%)
Clinical diagnosis stress urinary incontinence (no urodynamics performed)		20 (4.5%)	5 (3.3%)
Previous gynaecological surgery		146 (33.0%)	39 (25.5%)
Type of procedure	Ajust mini-sling	51 (11.5%)	11 (11.6%)
	Altis mini-sling	174 (39.4%)	25 (26.3%)
	Retropubic tape	95 (21.5%)	31 (32.6%)
	Obturator tape	120 (27.1%)	27 (28.4%)
	Autologous fascial sling	0 (0.0%)	1 (1.1%)
	MiniArc mini-sling	2 (0.5%)	0 (0.0%)
Received previous gynaecology surgery		442 (99.8%)	95 (62.1%)

^a^ Inter-quartile range

^b^ Standard deviation

^c^ International Consultation on Incontinence Questionnaire-Urinary Incontinence Short Form

d Mixed urinary incontinence defined as involuntary urinary leakage associated with physical exertion due to increased intrabdominal pressure as well as urgency []

### HCST responses

There was a high level of agreement between the positive/negative HCST (Yes/No question) and the amount of leakage reported on the schematic diagram (Table 2). With the example of baseline data, all of the 433 participants who said 'Yes' to experiencing leakage in the first question (positive HCST) made a response of either 'Small, Moderate, or Large' in the amount of leakage in the second question. Across all timepoints (*n* = 1063 observations), there were only three incidents of disagreement.

**Table 2:** Comparison of responses between the positive vs negative values (yes/no question) and the leakage amount on the HCST^a^

Leakage amount on HCST diagram	Total (across timepoints)	Baseline	1 year	2 years	3 years
Negative HCST^b^					
None	442	10 (100.0%)	159 (100.0%)	150 (99.3%)	123 (100.0%)
Small	0	0 (0.0%)	0 (0.0%)	0 (0.0%)	0 (0.0%)
Moderate	1	0 (0.0%)	0 (0.0%)	1 (0.7%)	0 (0.0%)
Large	0	0 (0.0%)	0 (0.0%)	0 (0.0%)	0 (0.0%)
Total (across leakage amounts)	443	10	159	151	123
Positive HCST^b^					
None	2	0 (0.0%)	1 (1.4%)	1 (1.5%)	0 (0.0%)
Small	148	47 (10.9%)	37 (52.9%)	39 (60.0%)	25 (48.1%)
Moderate	180	136 (31.4%)	17 (24.3%)	13 (20.0%)	14 (26.9%)
Large	426	250 (57.7%)	15 (21.4%)	12 (18.5%)	13 (25.0%)
Total (across leakage amounts)	620	433	70	65	52

^a^ Participants decided on the amount of leakage (None/Small/Moderate/Large) on data collection sheets. This was based on schematic diagrams provided at the time

^b^ Home Continence Stress Test

### Reliability

The Cronbach's alpha values (see Table 3) comparing the positive/negative values of the HCST performed 24 h apart were above 0.7. At all follow-up intervals, Cronbach's alpha was > 0.9.

**Table 3:** The consistency of the HCST responses^a^

	Positive vs negative value			Leakage amounts		
	α^b^	95% CI^c^	*N*^d^	α^b^	95% CI^c^	*n*
Baseline	0.74	(0.69–0.79)	436	0.88	(0.86–0.90)	437
1 year	0.92	(0.89–0.94)	217	0.95	(0.93–0.96)	176
2 years	0.91	(0.89–0.94)	204	0.95	(0.93–0.96)	145
3 years	0.91	(0.88–0.94)	171	0.94	(0.92–0.96)	122

^a^ Cronbach’s alpha values for the repeatability of the HCST test performed 24 hours apart for the a) positive vs negative value question (on a Yes/No question), and b) the leakage amount question. Participants were asked whether they leaked with the options to respond with Yes or No. Leakage amounts refer to the second HCST question with the following option to rate the amount of leakage None, Small, Moderate, or Large

^b^ Cronbach’s alpha (α)

^c^ 95% confidence intervals

^d^ These values are lower than that seen in the crosstabulation tables between positive vs. negative responses and that of the leakage amount responses or used in the later analysis because internal consistency was assessed before discrepancies between the HCST were addressed and when responses from the HCST performed after the pad test was used to fill in missing responses from the first HCST performed

The Cronbach's values for the reporting of severity of leakage amount 24 h apart were all > 0.87.

### Diagnostic accuracy

The comparison of the HCST to the pad test (for positive/negative values) showed a sensitivity range from 0.81–0.95 (see Table 4). The specificity ranged from 0.76–0.79. The best performing of the validity parameters was that of the NPV (0.96–0.99). However, the PPV was less, ranging from 0.32–0.97. The main drivers for low PPV were in the follow-up population (0.32–0.43), who by definition are different in nature compared to baseline due to the SUI surgical treatment received. The positive and negative likelihood ratios at the follow-up points show the HCST to be good at both ruling in and ruling out, with LR + values > 3 and LR − values < 0.25. This indicates a participant with a positive 24-h pad test is more than 4 times likely to have a positive HCST than a participant with a negative 24-h pad test, while a participant with a negative 24-h pad test is more than 4 times likely to have a negative HCST than a participant with a positive 24-h pad test. The follow-up AUC values of > 0.8 show the HCST to have high discriminative power. The value of 0.49 at baseline can be explained by 97% of the population reporting being incontinent at baseline on the reference standard 24-h pad test, and with only 12 continent participants high specificity is difficult to achieve.

**Table 4:** Validity for the positive/negative HCST compared to 24-h pad test^a^^,l^

	Baseline		1 year		2 years		3 years	
	Estimate	95% CI^b^	Estimate	95% CI^b^	Estimate	95% CI^b^	Estimate	95% CI^b^
Sensitivity	0.97	(0.96, 0.99)	0.81	(0.68, 0.94)	0.83	(0.67, 0.98)	0.95	(0.86, 1.00^ g^)
Specificity	0.00^e^	(0.00, 0.00^f^)	0.78	(0.72, 0.84)	0.76	(0.69, 0.82)	0.79	(0.72, 0.86)
PPV^c^	0.97	(0.95, 0.99)	0.43	(0.32, 0.55)	0.32	(0.20, 0.43)	0.43	(0.29, 0.58)
NPV^d^	0.00^ h^	(0.00, 0.00^f^)	0.95	(0.92, 0.99)	0.97	(0.94, 1.00)	0.99	(0.97, 1.00^ g^)
Odds ratio^i^	1.44	(0.08, 26.01)	15.49	(6.33, 37.95)	14.83	(4.77, 46.10)	74.62	(9.56, 582.23)
Positive LR^j^	0.97	(0.96, 0.99)	3.74	(2.72, 5.15)	3.41	(2.46, 4.72)	4.51	(3.16. 6.42)
Negative LR	0.70	(0.04, 0.99)	0.20	(0.10, 0.39)	0.23	(0.09, 0.56)	0.11	(0.02, 0.76)
AUC^k^	0.49		0.80		0.79		0.87	

^a^The positive value of the HCST compared to the positive value on the 24-h pad test (< 8 g vs ≥ 8 g) at all individual timepoints

^b^ 95% confidence intervals

^c^ Positive predictive value

^d^Negative predictive value

^e^ This specificity with a value of 0.00 reflects that there were 0 cases of a negative HCST among the negative pad tests. This was before the intervention in the SIMS trial [x] so likely to see less cases

^f^ The confidence intervals of 0.00 or 1.00 automatically creates an interval of the same value rather than genuine intervals

^g^ The upper confidence interval created was greater than 1.00 but because it is a proportion hence all values are constrained to 0.00–1.00

^h^ This NPV with a value of 0.00 reflects that there were zero cases of a positive pad test that had a negative HCST value, rather than a genuine value of 0.00

^i^ Diagnostic odds ratio

^j^ Likelihood ratios

^k^ Area uder the curve, according to sensitivity plotted against 1-specificity

^l^A post-hoc sample size calculation on the precision of the confidence interval around the estimated sensitivity or specificity was calculated; and with 443 participants, there is 90% power that the maximum width of the confidence interval around the estimated diagnostic accuracy measure is 0.1319

### Model to compare HCST to pad weights

There was a relationship between more severe leakage amounts on HCST and heavier pad weights (see Table 5). Those reporting “Small” leakage had pads 6 g heavier (6.23; 95% CI −1.65, 14.13) compared to the “None” leakage group. The difference for “Moderate” leakage was (7.62; 95% CI −1.30, 16.54). Therefore, neither of these groups showed a significant difference. The pad weight for those reporting “Large” leakage was significantly heavier by 28 g (28.07; 95% CI 7.40, 48.74). The subgroup analysis results were also reported (see Appendix B5 and B6).

**Table 5:** The difference in the 24-h pad test weights between the HCST severity groups

Parameter	Estimate^c^ (grams)	*P*-value	Confidence intervals
Unadjusted model			
None (ref.^a^)			
Small	6.42	0.11	(−1.49, 14.33)
Moderate	9.32	0.04	(0.42, 18.22)
Large	28.07	0.01	(7.40, 48.74)
Adjusted model^b^			
None (ref^a^)			
Small	6.23	0.12	(−1.65, 14.13)
Moderate	7.62	0.09	(−1.30, 16.54)
Large	24.46	< 0.01	(15.35, 33.56)

^a^The None group for leakage amounts was used as the reference category

^b^The adjusted model accounts for age, BMI, parity, previous gynaecological surgery and PFMT

^c^Measured in grams for the weight of pads

### Response to change

A “Small improvement” on the HCST had increased odds of a successful patient-reported outcome (Very/much improved) on PGI-I (see Table 6) [19]. This was not significant (OR 1.27; 95% CI 0.60, 2.71), whereas a “Big improvement” on the HCST (a reduction of ≥ two leakage groups) had a significantly higher odds of successful patient-reported outcome on PGI-I (OR 4.38; 95% CI 2.31, 8.31). There was a reduced odds of experiencing improved symptoms when the HCST gets worse (for example from Small to Moderate on the HCST diagram) (OR 0.44; 95% CI 0.07, 2.69).

**Table 6:** Logistic regressions for PGI-I according to the leakage amount change on the HCST compared to baseline^a^

Parameter^b^	OR^e^	P-value	Confidence intervals
Unadjusted model^c^			
No change (ref.^d^)			
Small improvement	1.27	0.53	(0.60, 2.71)
Big improvement	4.38	< 0.05	(2.31, 8.31)
Gets worse	0.44	0.37	(0.07, 2.69)

^a^PGI-I is treated in a dichotomous manner by treating 'Very much better' and 'Much better' as = 1, and the remaining answers ('A little better', 'No change', 'A little worse', 'Much worse' and 'Very much worse') = 0

^b^ The changes in HCST’s leakage severity is coded as 'Got worse' = between baseline & the timepoint the amount of leakage has got worse by any amount; 'No change' = the amount of leakage is the same; 'Small improvement' = amount of leakage group improved by 1 (for example from Large to Moderate or Small to None); and 'Big improvement' = amount of leakage improves by > 1

^c^ Timepoint is adjusted for to account for repeated observations

^d^ No change is the reference category

^e^ Odds ratio

## Discussion

### Summary of main results

The responders to the HCST showed a good understanding across the two questions of the HCST, with minimum conflicting values.

The Cronbach’s alpha analysis revealed strong consistency between the two sets of HCST results repeated 24 h apart (> 0.7). This allowed the measurement error to be calculated for more rigorous study. It was an important confirmation of reliability, since a measure is only precise if it remains consistent across repeated measures.

The comparison of diagnostic accuracy between the HCST to the 24- hour pad test (for positive/negative values), had recurring high levels of sensitivity, specificity, and NPV to show diagnostic accuracy. The high sensitivity indicates when SUI is present; the majority of cases were detected. The > 0.7 specificity credibly shows that the HCST identified those without SUI. The NPV, representing the identification of negatives, was the strongest parameter of HCST diagnostic accuracy.

In contrast, the PPV had lower results (across the timepoints except at baseline). The HCST would therefore be at risk of introducing false positives. There are a number of potential explanations to this poor PPV. Both the PPV and NPV can be influenced by the prevalence of a condition. The data for this study was from a RCT [17] of surgical treatment for SUI, where the objective success rate was reported in average of 80% at all follow-up time points [20]. Therefore, as expected, there was a low SUI prevalence at all the follow-up time points which can explain the low PPV. This supported by the fact that when SUI was prevalent at baseline, the PPV is 0.97 and then dramatically fell at all follow-up points (i.e., once participants have been treated). Since the diagnostic threshold influences what is considered disease, and the subsequent prevalence, a lower diagnostic threshold on the pad test therefore may influence the PPV. Lastly, there is a strong possibility that the PPV appears low because the HCST is a more sensitive assessment tool compared to the 24-h pad test. A recent randomised study suggested that the clinician-observed CST is more sensitive than the 24-h pad test [7] when compared to urodynamics. Similar findings have been reported elsewhere [17].

In the adjusted model, those reporting Moderate and Large leakage on the HCST diagram had significantly heavier pads on the 24-h pad test. The two tools therefore shared a good level of agreement. The pad weights increased further for those with moderate to severe urgency (see Appendices B5 and B6). This relationship was further examined by studying the association between moderate to severe urgency perception and a positive HCST (see Appendix B7). The HCST has a heighted sensitivity among those experiencing moderate and severe urgency. It is well documented that women with severe urgency report worse impact on their QoL [27].

A 'Small improvement' (≤ 1 group) on the HCST was not associated with reports of 'Much better/Very much better' symptoms on the PGI-I (successful outcome). This is in keeping with how ‘A little better’ on the PGI-I scale was not considered as a successful outcome post-surgery. However, a 'Big improvement' (≥ 2 groups) was significantly associated with a successful outcome on PGI-I. The HCST has therefore been able to detect the participants’ reported success on PGI-I.

### Strengths and weaknesses

In addition to the HCST’s diagnostic power, many of the personal and financial issues faced by the existing measurements are addressed. HCST provides the convenience of both a pad-free test and being performed at home. The HCST therefore avoids hospital visits, the use of extra healthcare resources, and eliminates healthcare professional bias in reporting the outcome of interventions. These strengths could potentially translate to improved recruitment and retention in SUI trials. Despite most COVID restrictions being lifted, reduced footfall in hospitals is still a valued attribute seen with the HCST [28].

The method of standardisation, ‘comfortably full bladder’, for the HCST in the SIMS RCT [20] has been documented as the method of standardisation for the CST that has the highest specificity [7]. Nevertheless, the lack of objective standardisation of the bladder volume at the start of the test can be a potential limitation.

Conventionally, a new assessment tool being introduced has its diagnostic accuracy compared to the ‘standard’, or the most reliable available method. It could be argued this would be urodynamic testing in this scenario. A key limitation of the HCST in this evaluation was the low PPV, the reasons of which have been discussed earlier.

### Current context

The HCST is a novel assessment tool, however in some respects it can be perceived as the ‘self-reported version’ of the CST. In other domains of gynaecology and wider medicine, there has been a noticeable shift to self-testing alternatives with the example of self-collected cervical smear samples [29]. If the same efforts are made to ensure trial follow-up measurements are more convenient for participants, then trials are equally expected to see improved retention. Patient self-assessment studies have produced reliable measurement in other fields of medicine despite initial hesitation and concern [30, 31].

### Implications

Further evaluation of the HCST is required in clinical practice, i.e. in the population presenting to the incontinence clinics. Prediction models combining HCST results and responses to symptom severity questionnaires is an area of further research. Qualitative research to determine whether participants favour the HCST to the other alternative assessment tools would also be of value.

## Conclusion

The HCST is a valid and reliable patient-reported objective assessment tool of SUI in surgical clinical trials with good specificity, sensitivity, and consistency that eliminates the need for hospital visits and assessor’s bias. HCST can replace the 24-h pad tests in future trials. Further research is required to see if it translates to better retention in clinical trials and before it can be used in standard clinical practice.

## Supplementary information

Below is the link to the electronic supplementary material.

Supplementary file1 (DOCX 500 KB)
