# Supplementary material for: The “Aberdeen Home Continence Stress Test”: a novel objective assessment tool for female stress urinary incontinence
Source: Int Urogynecol J. 2023 Apr 13;34(8):1961–9. doi: 10.1007/s00192-023-05530-4 (PMC10415466; doi:10.1007/s00192-023-05530-4)
Supplement: Supplementary file 1 — Supplementary file1 (DOCX 500 KB) [file 192_2023_5530_MOESM1_ESM.docx]

**Appendices**


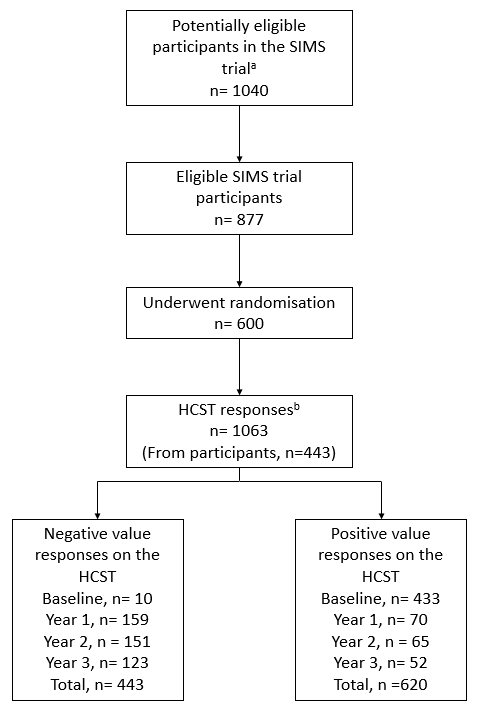


**Appendix B1 – Flow diagram from the participants in the original SIMS trial to the HCST responses collected from the trial.** (Created on Powerpoint Microsoft).
^a^Data came from the Single-Incision Mini-Slings randomised control trial, as referenced in the main paper.
^b^From here and below on the flow diagram, the responses (across four time points) are being reported rather than the individuals at baseline as reported above this point.
At year 1 follow-up point, the response rate was 52% compared to baseline.
At year 2 follow-up point, the response rate was 49% compared to baseline.
At year 3 follow-up point, the response rate was 40% compared to baseline.


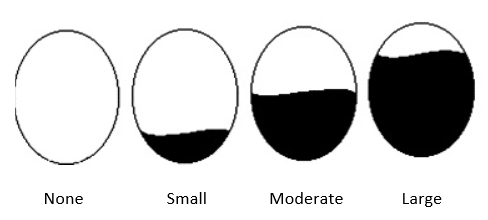

**Appendix B2 – Schematic diagram participants received in the original trial to answer the second leakage amount question.** (Created on Paint).
None represents none or only a few drops of leakage. Small represents 1/3 of the tissue paper saturated, as assessed by the participants. Moderate represents 1/2 of the tissue paper saturated, as assessed by the participants. Large represents 2/3 of the tissue paper saturated, as assessed by the participants.


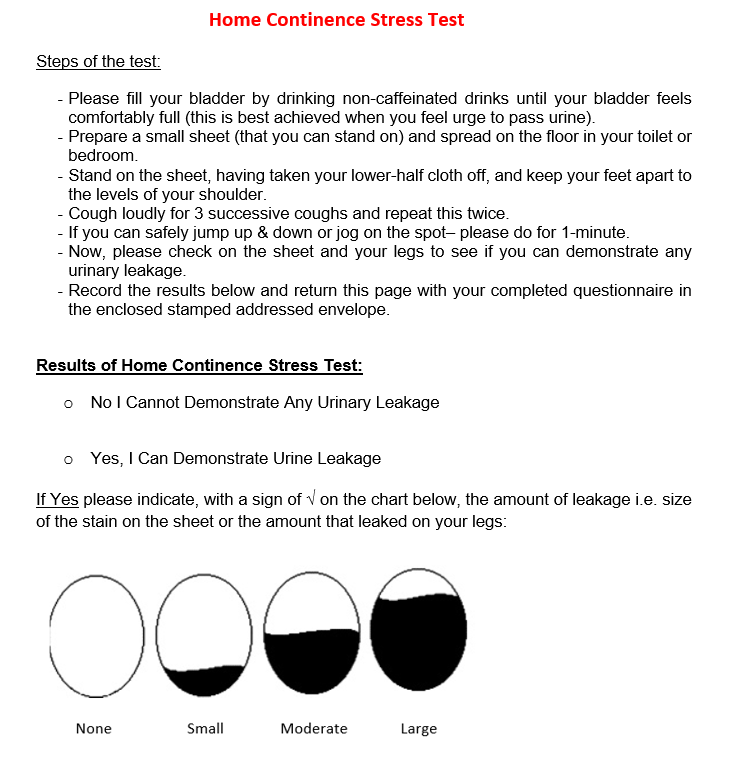


**Appendix B3 – Instructions for using the HCST^a^ as provided to the participants in the SIMs^b^ trial.**^a^Home Continence Stress Test
^b^Single-Incision Mini-Slings

**Appendix B4 – Cleaning data for data management** (Created on Powerpoint Microsoft).

**
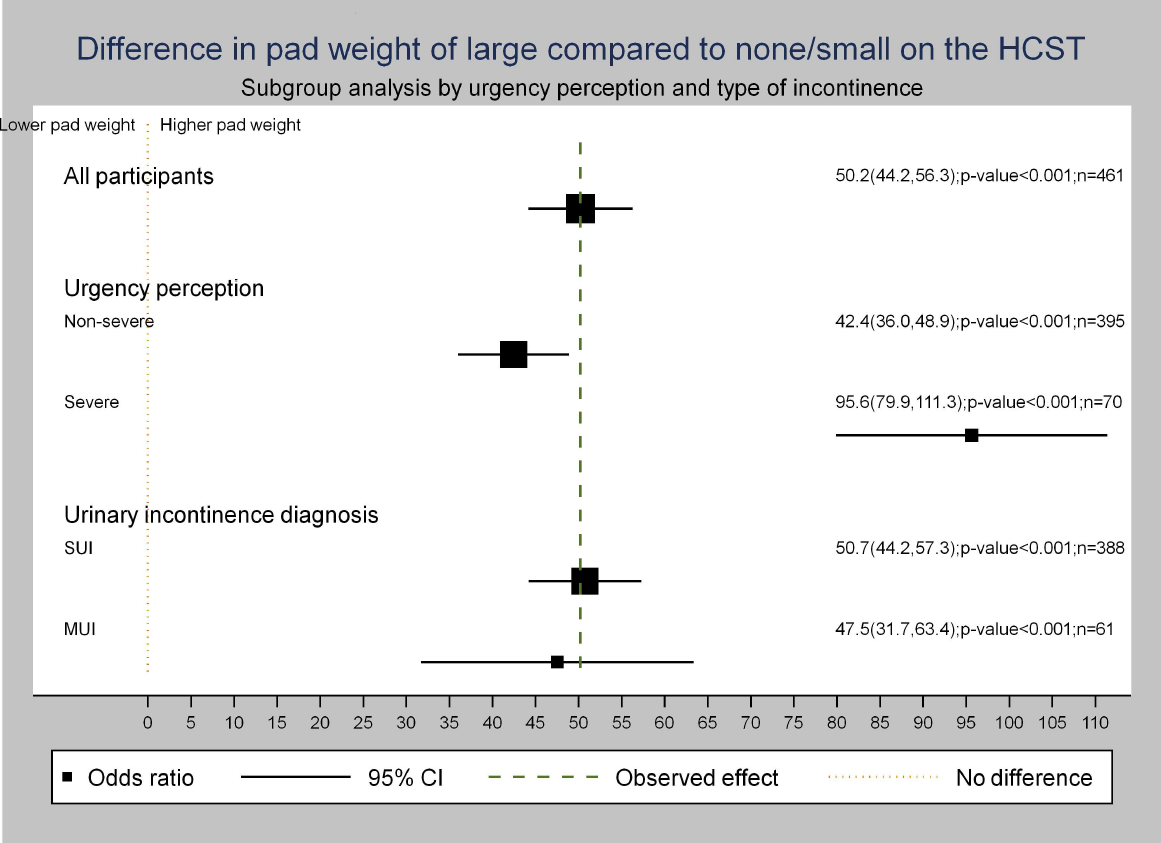
**

**Appendix B5 – Subgroup analysis among Moderate leakage reported.**Stress Urinary Incontinence (SUI) is defined as involuntary leakage on physical exertion due to increased abdominal pressure.
Mixed Urinary Incontinence (MUI) defined as involuntary urinary leakage associated with physical exertion due to increased intrabdominal pressure as well as urgency.

**
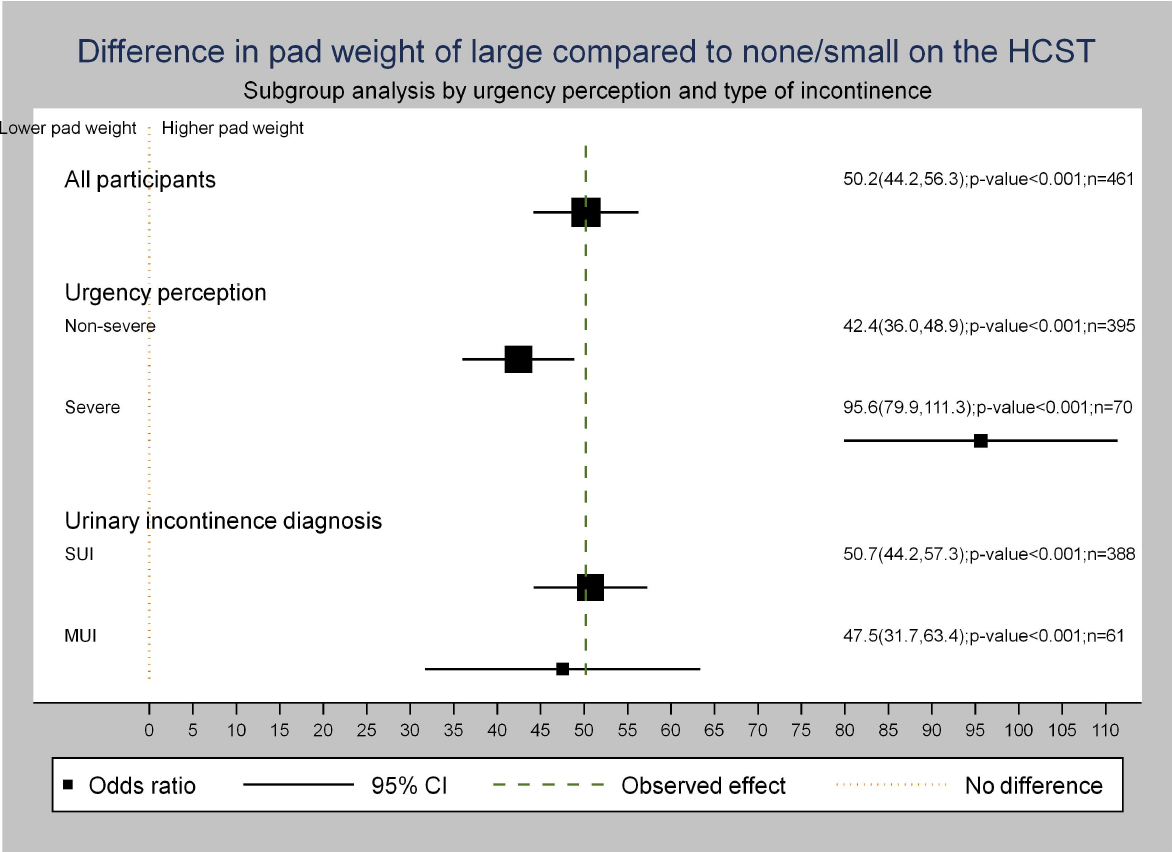
**

**Appendix B6 – Subgroup analysis among Large leakage reported.**Stress Urinary Incontinence (SUI) is defined as involuntary leakage on physical exertion due to increased abdominal pressure.
Mixed Urinary Incontinence (MUI) defined as involuntary urinary leakage associated with physical exertion due to increased intrabdominal pressure as well as urgency.

**Appendix B7 – Association (odds) between urgency perception and a positive HCST**

| **Parameter** | **OR^a^** | **P-value** | **Confidence intervals** |
| --- | --- | --- | --- |
| **Adjusted model^b^** |  |  |  |
| None/mild (ref^c^) |  |  |  |
| Moderate/severe | 2.06 | 0.01 | (1.23, 3.45) |
| **Unadjusted model** |  |  |  |
| None/mild (ref.^c^) |  |  |  |
| Moderate/severe | 2.10 | <0.01 | (1.30, 3.39) |

^a^Odds ratio
^b^The adjusted model accounts for age, BMI, parity, previous gynaecological surgery and PFMT. ^c^None/mild urgency perception was used as the reference category.
